# Placental Lactogen Is Expressed but Is Not Translated into Protein in Breast Cancer

**DOI:** 10.1371/journal.pone.0087325

**Published:** 2014-01-24

**Authors:** Traci R. Tuttle, Eric R. Hugo, Wilson S. Tong, Nira Ben-Jonathan

**Affiliations:** Department of Cancer Biology, University of Cincinnati College of Medicine, Cincinnati, Ohio, United States of America; Sun Yat-sen University Medical School, China

## Abstract

**Introduction:**

Several studies reported that the pregnancy-specific hormone placental lactogen (hPL) is expressed at both mRNA and protein levels in breast cancer. The overall objective was to establish hPL, the product of the *CSH1* and *CSH2* genes, as a biomarker for breast cancer.

**Methods:**

CSH expression was determined at the mRNA level in breast cancer cell lines (BCC) and primary carcinomas by real-time and conventional PCR and the products verified as CSH1 by sequencing. Expression of hPL protein was examined by western blots and immuno-histochemistry, using commercial and custom-made polyclonal and monoclonal antibodies.

**Results:**

Variable levels of CSH mRNA were detected in several BCC, and in some primary tumors. We detected a protein, slightly larger than recombinant hPL by western blotting using several antibodies, leading us to postulate that it represents an hPL variant (‘hPL’). Furthermore, some monoclonal antibodies detected ‘hPL’ by immunohistochemistry in breast carcinomas but not in normal breast. However, further examination revealed that these antibodies were non-specific, as efficient suppression of CSH mRNA by shRNA did not abolish the ‘hPL’ band. Custom-made monoclonal antibodies against recombinant hPL detected hPL of the correct size in placental lysate and hPL-overexpressing BCC, but not in unmodified cells or primary carcinomas. hPL protein was detected only when mRNA was increased several thousand fold.

**Conclusions:**

We call into question previous reports of hPL expression in breast cancer which relied on mRNA levels as surrogates for protein and/or used improperly validated antibodies to measure hPL protein levels. Our data suggests that an inhibitory mechanism(s) prevents translation of *CSH* mRNA in breast cancer when not highly expressed. The mechanism by which translation of CSH mRNA is inhibited is intriguing and should be further investigated.

## Background

Human placental lactogen (hPL), also known as chorionic somatomammotropin hormone (CSH), is 22-kDa protein member of the family of human lactogens, which also includes prolactin (hPRL) and growth hormone (hGH). The three lactogens have a similar 3D structure, and all bind to and activate the prolactin receptor (PRLR) [1]. Unlike hPRL and hGH, which are produced primarily by the pituitary, hPL is produced by the syncytiotrophoblast of the placenta, and is found at very high levels in the maternal circulation during mid to late pregnancy [2].

Two genes, *CSH1* and *CSH2*, encoding for an identical hPL protein, are part of the GH gene cluster on chromosome 17. This cluster also includes pituitary GH (*GH1*), a placental GH variant (*GH2*), and a CSH-like gene (*CSHL1*), thought to be a pseudo-gene. The genes in this cluster have evolved through a series of duplication events and show greater than 90% sequence identity in their coding and immediate flanking regions [3]. Tight regulation at the transcriptional level ensures that *GH1* is specifically expressed in the pituitary, while *CSH1, CSH2* and *GH2* are expressed only in the placenta. Expression of the *CSH* genes is under the control of transcriptional enhancer sequences in the 3′ regions, a pituitary specific repressor sequence, and a locus control region located 15–30 kb upstream of the cluster [4]–[6]. However, there is little knowledge of the translational control of hPL.

Choriocarcinomas are tumors that primarily arise in placental tissue, and can also form in ovaries, testis and other tissues. Several choriocarcinoma cells lines, e.g., BeWo, JAR and JEG3, have been used to examine the regulation of *CSH* expression. This was primarily done by employing transient transfection with promoter and enhancer sequences of CSH, driving expression of reporters such as luciferase [7], [8]. While many studies found expression of endogenous *CSH* gene in such cell lines, most failed to identify hPL protein production [7], [9]–[11], raising the possibility that the *CSH* gene is not translated into protein in these cell lines.

Expression of hPL was also reported in breast, ovarian and testicular cancers [12]–[14]. Older studies detected hPL protein in breast tumors and in serum from women with breast cancer [15], [16]. One study found that presence of hPL in breast tumors negatively correlated with patient survival [15], while another study did not detect hPL in serum from breast cancer patients [17]. More recently, the *CSH* genes were reported to be amplified in breast tumors, and this was correlated with aneuploidy, lymph node metastases and overexpression of the Her2/neu oncogene [18]; detection of hPL in tumors by immunohistochemistry (IHC) correlated with gene amplification. Among studies that examined normal breast tissue, only one reported detectable hPL protein, which was not confirmed at the mRNA level, as was done for hPRL and hGH in the same study [19].

Given the above reports, our main objective was to explore whether hPL can serve as a biomarker for breast cancer. To this end, we compared expression of hPL mRNA and protein in breast cancer cell lines (BCC), normal breast tissue, primary breast tumors, and choriocarcinoma cell lines, using complementary approaches that include conventional and real-time PCR, western blotting, IHC, overexpression and knockdown. Collectively, our data lead us to conclude that hPL is expressed, but is not translated into protein in breast cancer. This raises a cautionary note for previous studies that rely exclusively on gene expression without confirmation at the protein levels. We also emphasize the need for a vigorous validation of any antibodies used in western blotting or IHC to verify expression of hPL proteins. Finally, we speculate about potential mechanisms which suppress the translation of CSH mRNA in cancer cells and suggest that these should be an interesting subject of future investigation.

## Methods

### Ethics Statement

This study, which involved archived tumor samples but not direct patient participation, has been approved by the University of Cincinnati Institutional Review Board (IRB).

### Tissues, cell lines and recombinant proteins

Fresh frozen or formalin-fixed, paraffin embedded (FFPE) normal breast tissue, primary breast carcinomas and placentas were obtained from Asterand (Detroit, MI), and from University of Cincinnati Cancer Institute Tumor Bank. Breast cancer cell lines T47D, MCF7, ZR75, MDA-MB-231 (MB231) and MDA-MB-468 (MB468), non-tumorigenic breast epithelial cell lines MCF10a and HME, the choriocarcinoma cell lines JAR and BeWo and human embryonic kidney cell line HEK293 were obtained from American Type Culture Collection (Manassas, VA). Recombinant hPL, hPRL and hGH were obtained from Protein Laboratories (Rehovot, Israel)

### Antibodies

The following polyclonal antibodies were used: two rabbit polyclonal antibodies (Poly-1 and Poly-2) raised against recombinant hPL (rhPL) for our laboratory by Protein Laboratories (Rehovot, Israel), a rabbit polyclonal antibody (Poly-3), raised against purified hPL from human placenta, provided by Dr. Stuart Handwerger (Cincinnati Children's Hospital Medical Center), and a sheep polyclonal antibody (Sheep) raised against placental-purified hPL (Aviva, San Diego, CA). The following monoclonal antibodies were used: a mouse monoclonal antibody MAB5757 (mAb-1), raised against rhPL, from R&D Systems (Minneapolis, MN), two mouse monoclonal antibodies (mAb-4 and mAb-6), raised against rhPL, produced for our laboratory by Protein Laboratories, and a mouse monoclonal (mAb-12), raised against a synthetic peptide corresponding to amino acids 130–140 of hPL, produced for our laboratory by Abmart (Shanghai, China). A rabbit polyclonal antibody raised against recombinant PRL was made and validated by our laboratory, and a rabbit polyclonal antibody raised against pituitary GH was obtained from the NIDDK. A monoclonal antibody against β-actin (A5441) was from Sigma-Aldrich (St. Louis, MO).

### Cell culture

T47D, MCF7, ZR75 and JAR cells were maintained in RPMI 1640 medium supplemented with 10% fetal bovine serum (FBS; Atlanta Biologicals, Lawrenceville, GA) and 0.01% Primocin antibiotic (Invivogen, San Diego, CA). T47D cell medium was supplemented with 5 ug/ml of human insulin (Sigma-Aldrich). MB231 and MB468 cells were maintained in DMEM with 1 g/L glucose supplemented with 10% FBS and 0.01% Primocin. MCF10a and HME cells were maintained in DMEM F:12 supplemented with 5% horse serum (Atlanta Biologicals) or 0.4% bovine pituitary extract respectively, and 5 ug/ml human insulin, 0.5 ug/ml hydrocortisone, 20 ng/ml human epidermal growth factor and 0.01% Primocin. BeWo cells were maintained in DMEM F:12 supplemented with 10% FBS and 0.01% Primocin. Hek293 cells were maintained in DMEM with 4.5 g/L glucose, 10% FBS and 0.01% Primocin.

### Cloning, lentiviral vector production and overexpression of hPL

The full length *CSH1* gene was cloned from T47D cells as well as human placenta, using AccuPrime Taq high fidelity polymerase (Invitrogen, Carlsbad, CA), using the primer pair F: ACAGAAACAGGTGGGGTCAAG, R: TTATTAGGACAAGGCTGGTGGG. The PCR products were amplified to 40 cycles, separated on a 1% agarose gel, purified with the GeneJET gel purification kit (Fisher Scientific, Waltham, MA), and confirmed by sequencing (Genewiz, South Plainfield, NJ). Products were cloned into the pCR8/GW/TOPO donor vector (Invitrogen) and was combined with the destination vector pLenti CMV Puro DEST (Addgene plasmid 17452) using LR Clonase (Invitrogen) to create pLentihPL [20]. Competent E-coli were transformed with pLentihPL, psPAX2, and pMD2.G plasmids. After selection of clones, plasmids were isolated using the GeneJet maxi-prep kit. HEK293 cells were transfected with pLentihPL plasmids using Fugene HD (Roche, Penzberg, Germany), and supernatants containing viral particles were collected at 24 and 48 h. MCF7, T47D, MB231 and MB468 cells were infected with the recombinant lentivirus and cultured for two weeks in puromycin selection media before harvesting of total RNA and proteins.

### Suppression of hPL expression with shRNA

SureSilencing shRNA for *CSH* and scrambled control plasmids were purchased from Qiagen (Hilden, Germany). Competent E-coli were transformed with the plasmids, the clones were selected and the plasmids isolated using the GeneJet plasmid maxi-prep kit. Constructs were transfected into cells using Lipofectamine 2000 (Invitrogen). MCF7 and ZR75 cells were plated 18–24 hours prior to transfection, and were transfected 70% confluence with the shRNA constructs using Lipofectamine 2000. The transfected cells were selected with puromycin for two weeks prior to harvesting total RNA and proteins.

### Real-time and conventional PCR

RNA from tissues was isolated using TriReagent (Molecular Research Center, Cincinnati, OH) with agitation and tissue lysing using a TissueLyser (Qiagen). Total RNA was isolated using the illustra RNAspin Mini kit from GE Healthcare (Buckinghamshire, UK) and was reverse transcribed using the Maxima First Strand cDNA Synthesis kit from Fisher. The following intron-spanning primer sets were used for both real-time and conventional PCR: CSH (F: AAAAAGGGCCCACAAGAGAC, R: GGGGTCACAGGATGCTACTC, β-Actin (F: ATCTGGCACCACACCTTCTACA, R: ACAGCCTGGATAGCAACGTACA). Real-time PCR was performed on 30 ng of cDNA using Absolute Blue QPCR SYBR Green ROX mix from Thermo Scientific, on an Applied Biosystems StepOnePlus real-time PCR system. Cycle parameters for both real-time and conventional PCR were 95 C for 15 minutes for polymerase activation, followed by 95 C for 30 sec, 67 C for 15 sec and 72 C for 1 minute. After normalization for β-actin, changes in gene expression were calculated from cycle threshold measurements [21]. Conventional PCR was performed on 50 ng of cDNA using Immomix Red 2X solution (Invitrogen) and an Eppendorf Mastercycler. The PCR products were electrophoresed on 1% agarose gels and visualized using ethidium bromide.

### Western blot analysis

Cells were lysed in modified RIPA buffer (50 mM Tris pH 8.0, 150 mM NaCl, 1% NP40, 0.5% deoxycholate, 1% SDS and 5 mM EDTA) containing protease inhibitors (Fisher). Frozen tissues were ground with a mortar and pestle before lysing in the same buffer described above. Proteins were resolved on 15% SDS-PAGE and were transferred to polyvinylidene fluoride membranes. Membranes were blocked in Tris-buffered saline with 0.1% Tween (TBS-T) containing 0.5% dehydrated milk. Membranes were incubated with primary antibodies for 18–24 hours at 4 C, washed in TBS-T and incubated for 1 h in HRP-conjugated secondary antibody. Blots were developed on film using SuperSignal West Pico chemiluminescent substrate solution. Membranes were re-probed with β-actin antibody as a loading control. Recombinant proteins were obtained from Protein Laboratories (Rehovot, Israel), and placental lysate was purchased from BioChain (San Francisco, CA).

### Immunohistochemistry and immunocytochemistry

Staining for hPL was performed on FFPE normal breast tissues, primary breast carcinomas and placentae. Slides were deparaffinized and rehydrated through a graded ethanol series. Endogenous peroxidase activity was quenched with 3% hydrogen peroxide, and antigen retrieval for tissues was done by placing the slides in sodium citrate buffer, pH 6.0 for 10 min in 90–95 C. Slides were blocked for 2 h in PBS containing 1% BSA, 15% sheep serum and 5% normal human serum, followed by overnight incubation with primary antibodies at 4 C in a humidity chamber. After washing, slides were incubated with HRP-conjugated sheep anti-mouse secondary antibody (GE Healthcare) for 3 h. Color was then developed using ImmPact DAB substrate from Vector Laboratories. Slides were counterstained with Hematoxylin, washed, dehydrated and mounted. For immunocytochemistry, cells were plated onto chamber slides 24 h before fixation in 4% formaldehyde. The same procedure for IHC was followed, except for the hydration and dehydration steps.

### Data analysis

Each experiment was repeated 2–3 times. When applicable, data are presented as means±SEM.

## Results

### Breast cancer and choriocarcinoma cell lines express CSH mRNA at variable levels

**Figure 1:**
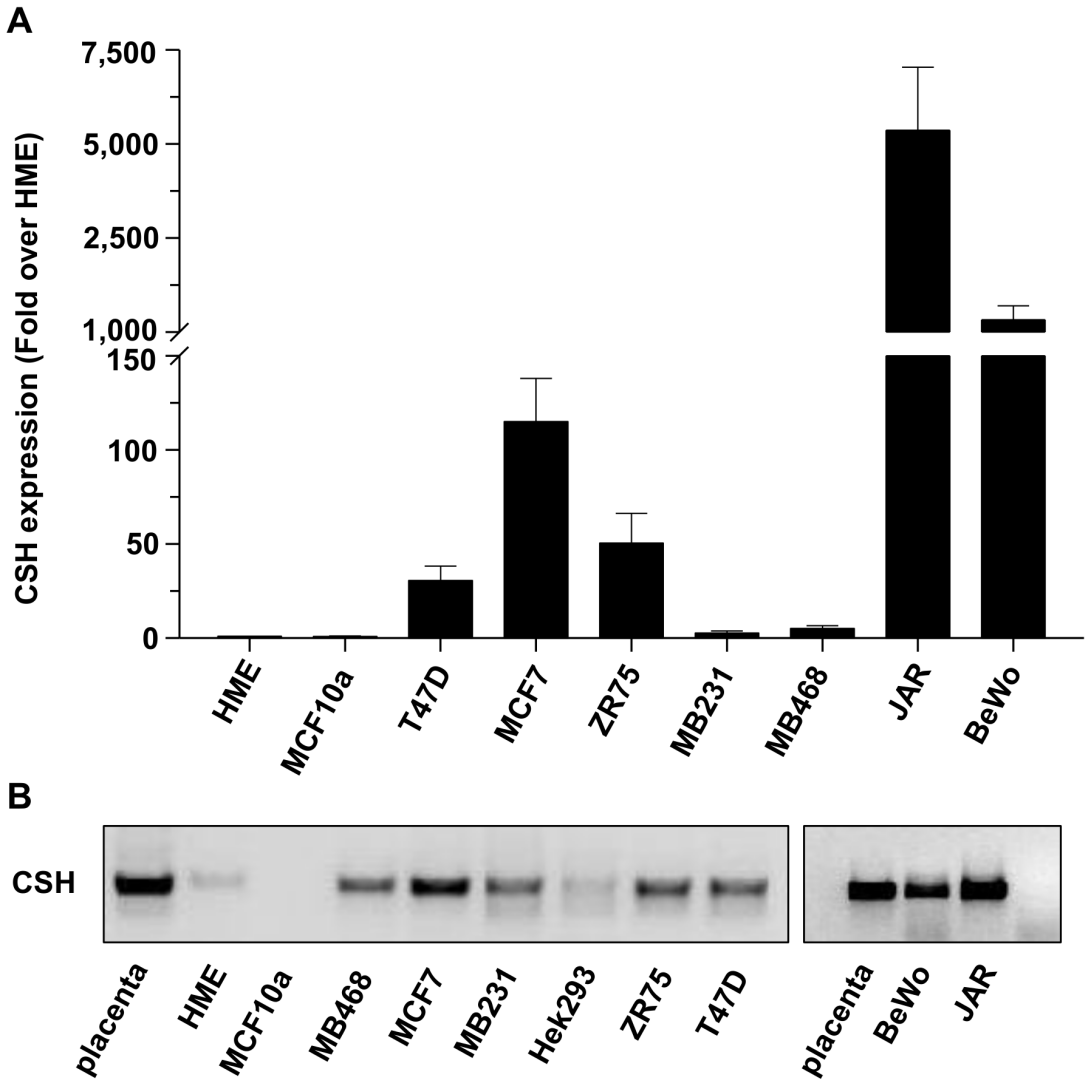
The CSH gene is expressed at variable levels in breast cancer and choriocarcinoma cell lines. (A) Expression of CSH mRNA in 5 breast cancer cells lines, 2 non-tumorigenic breast epithelial cells (HME and MCF10) and 2 choriocarcinoma cell lines (JAR and BeWo) as determined by real-time PCR. Data are expressed as fold change over HME after correction for β-actin (means±SEM; N = 3). (B) CSH products of identical sizes are produced in placenta, breast cancer and choriocarcinoma cell lines. The products were generated by using 30 cycles of conventional PCR, followed by electrophoresis on a 1% agarose gel and visualization with ethidium bromide.

Expression of the *CSH* genes was examined by real-time and conventional PCR in five breast cancer cell lines, two non-tumorigenic breast epithelial cell lines, two choriocarcinoma cell lines and human placenta. Because the primer set amplifies both *CSH1* and *CSH2* genes, products are referred to as *CSH*. Real time PCR revealed variable levels of *CSH* expression in BCC, with 20 to 100 fold higher expression levels seen in the two choriocarcinoma cell lines (Figure 1A). Expression of *CSH* was low to undetectable in the non-tumorigenic breast epithelial cell lines MCF10a and HME. Conventional PCR followed by gel electrophoresis confirmed that the products were of the correct size (Figure 1B). Note, however, the apparent lack of correlation in expression levels between real-time and conventional PCR as the latter method is only semi- quantitative. The full length PCR products from MCF7 and T47D cells as well as human placenta were gel purified and sequenced. The sequence of both products was confirmed to be *CSH1* variant 1 (NM_001317), although this does not preclude the possibility that *CSH2* (which generates an identical hPL protein) or other variants are also present, at extremely low levels.

### Detection of ‘hPL’ in breast cancer and choriocarcinoma cell lines by western blotting

**Figure 2:**
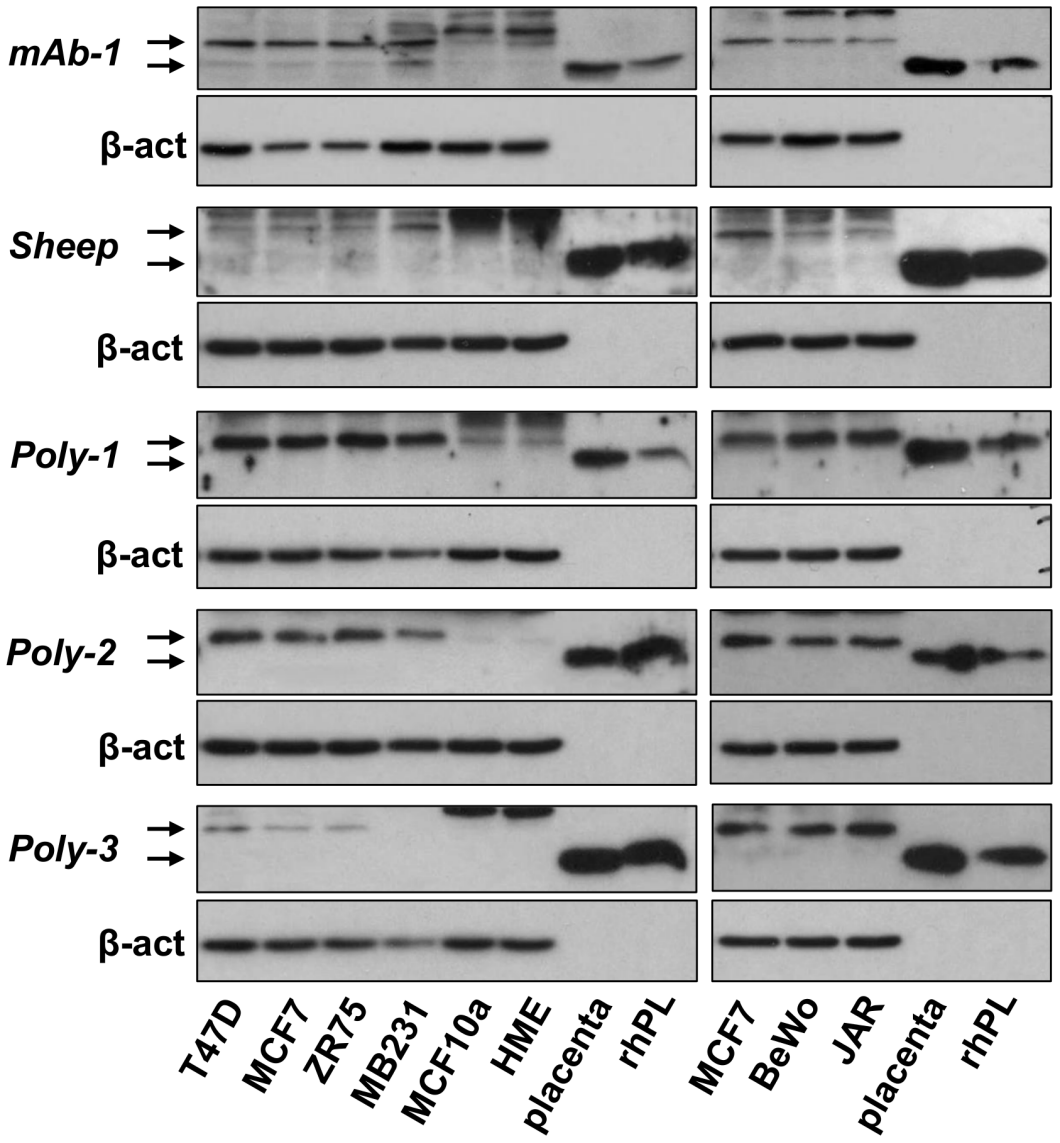
Five different antibodies detect an hPL-like protein (‘hPL’) in breast cancer and choriocarcinoma cell lines by western blotting. Whole cell lysates from four breast cancer, two non-tumorigenic breast epithelial, and two choriocarcinoma cells were subjected to western blotting. hPL was detected using commercially available (mAb-1 and Sheep), and custom-made (Poly-1, Poly-2 and Poly-3) antibodies. 40 ug of protein was loaded for each cell line; 0.5 ug of placental lysate and 2 ng rhPL were loaded as positive controls. β-actin served as a loading control.

The next objective was to determine whether the hPL protein is detectable in the above cell lines. Because only hPL purified from the human placenta was commercially available, we had recombinant hPL synthesized by Protein Laboratories (Rehovot, Israel) as a positive control for the various assays, and for the subsequent generation of polyclonal and monoclonal antibodies. Several polyclonal and monoclonal antibodies were obtained from different sources (see Materials and Methods), and we also generated two rabbit polyclonal antibodies against rhPL. As evident in Figure 2, all antibodies recognized rhPL and placental-derived hPL of the expected size (22 kDa). However, variable levels of a product, which appeared to be 2–3 kDa larger than placental or recombinant hPL, were detected by the same antibodies in both BCC and choriocarcinoma cells, leading us to believe that it represent a post-translationally modified hPL (‘hPL’).

### The protein recognized by hPL antibodies is neither hPRL nor hGH

**Figure 3:**
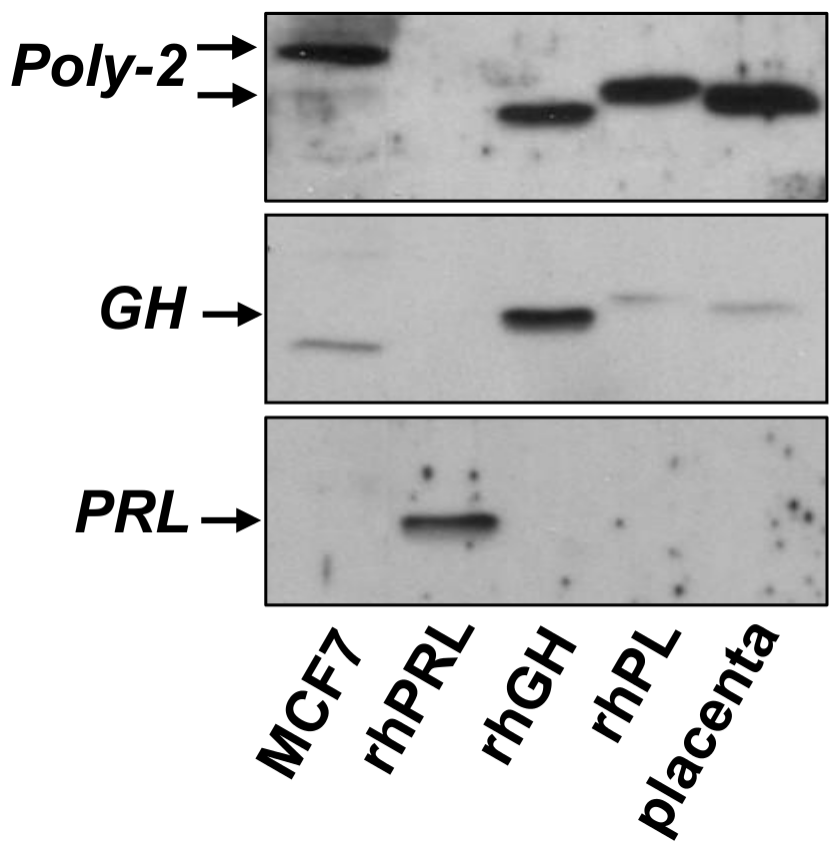
Antibodies for hGH and hPRL do not detect ‘hPL’. Western blots with 40 µg of MCF7 breast cancer cell lysate, 5 ng of recombinant hPL, hPRL and hGH and 0.5 µg of placental lysate were probed with antibodies for hPL, hPRL and hGH.

Because of the structural similarity of hPL to hPRL and hGH, we next examined whether either lactogen was detectable in breast cancer cell lysates by western blots. Using antibodies directed against hPRL and hGH and recombinant proteins as positive controls, PRL or GH were undetectable in MCF7 cell lysate (Figure 3). Although Poly-2 antibody against rhPL cross-reacted with rhGH, it appeared as a smaller protein than rhPL, unlike ‘hPL’ detected in breast cancer cells. The GH antibody recognized a protein much smaller than either rhPL or rhGH in breast cancer cell lysate that is likely non-specific. Neither GH nor PL antibodies cross-reacted with rhPRL (Figure 3).

### Detection of ‘hPL’ in some breast tumors but not in normal breast tissue by immunohistochemistry

**Figure 4:**
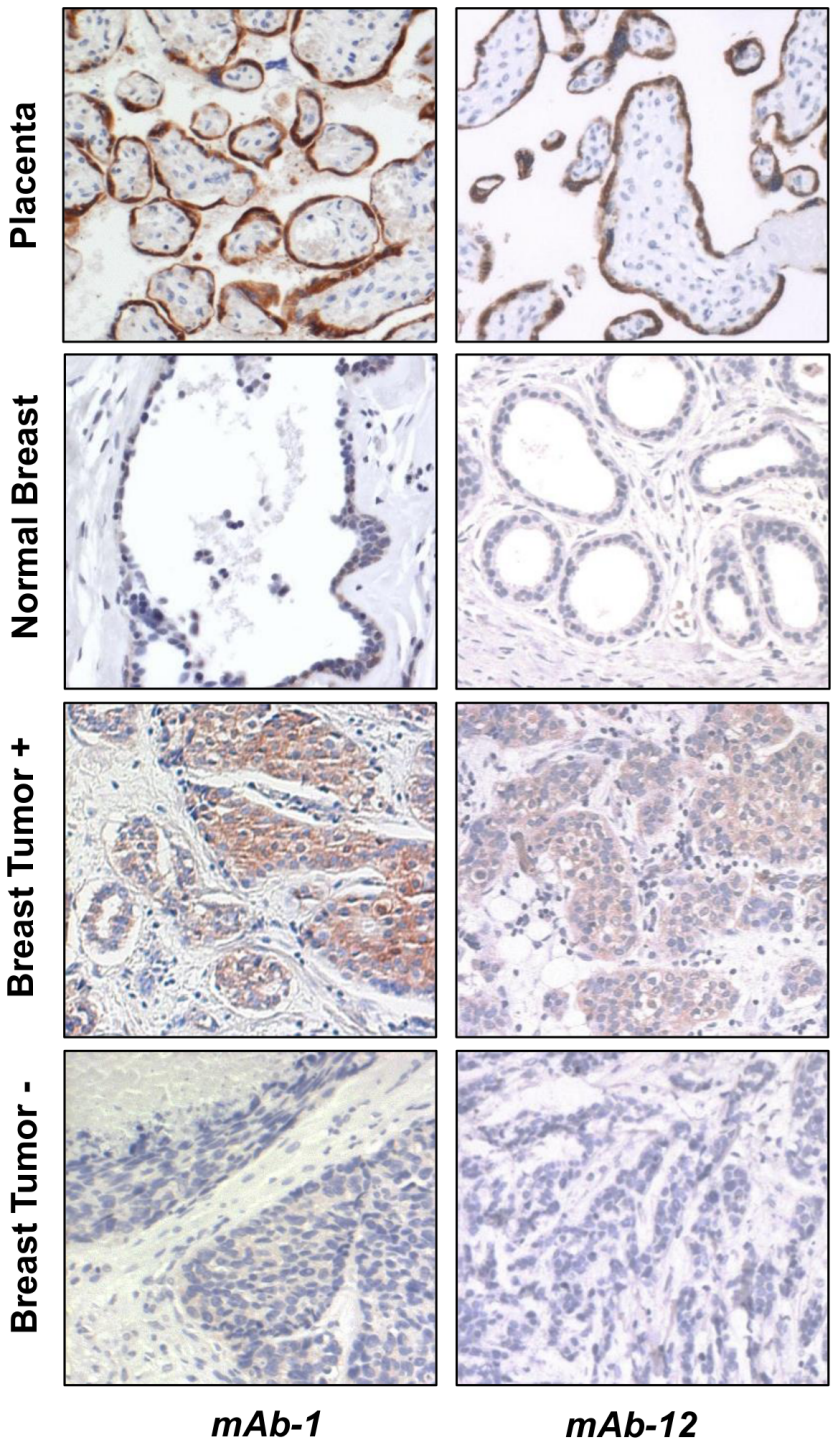
Immunohistochemical staining of human breast tumors using mAb-1 and mAb-12 monoclonal antibodies against hPL. Term placenta (top) showing specific staining of the syncytial layer by both antibodies. Representative examples of staining-negative normal tissue and staining-positive and -negative tumors are shown. All normal tissues tested were negative and some, but not all tumors, were positive.

Following the detection of ‘hPL’ in breast cancer cell lines by multiple antibodies, we next used mAb-1 and mAb-12 to determine whether immunoreactive hPL is detectable in primary breast tumors and normal breast tissue. Placental tissue, used as a positive control, confirmed that both antibodies heavily stained only the syncytiotrophoblast layer (Figure 4). Some, but not all, of the breast tumors showed positive staining, while none of the normal breast tissues tested were positive. Positive staining was evident only in the epithelial compartment and not in surrounding stromal cells, and was cytoplasmic and diffuse. Figure 4 shows examples of two normal breast tissues with negative staining, one breast tumor with a positive staining and another breast tumor with a negative staining.

### Failure to detect hPL in breast cancer or choriocarcinoma cells using our newly generated monoclonal antibodies

**Figure 5:**
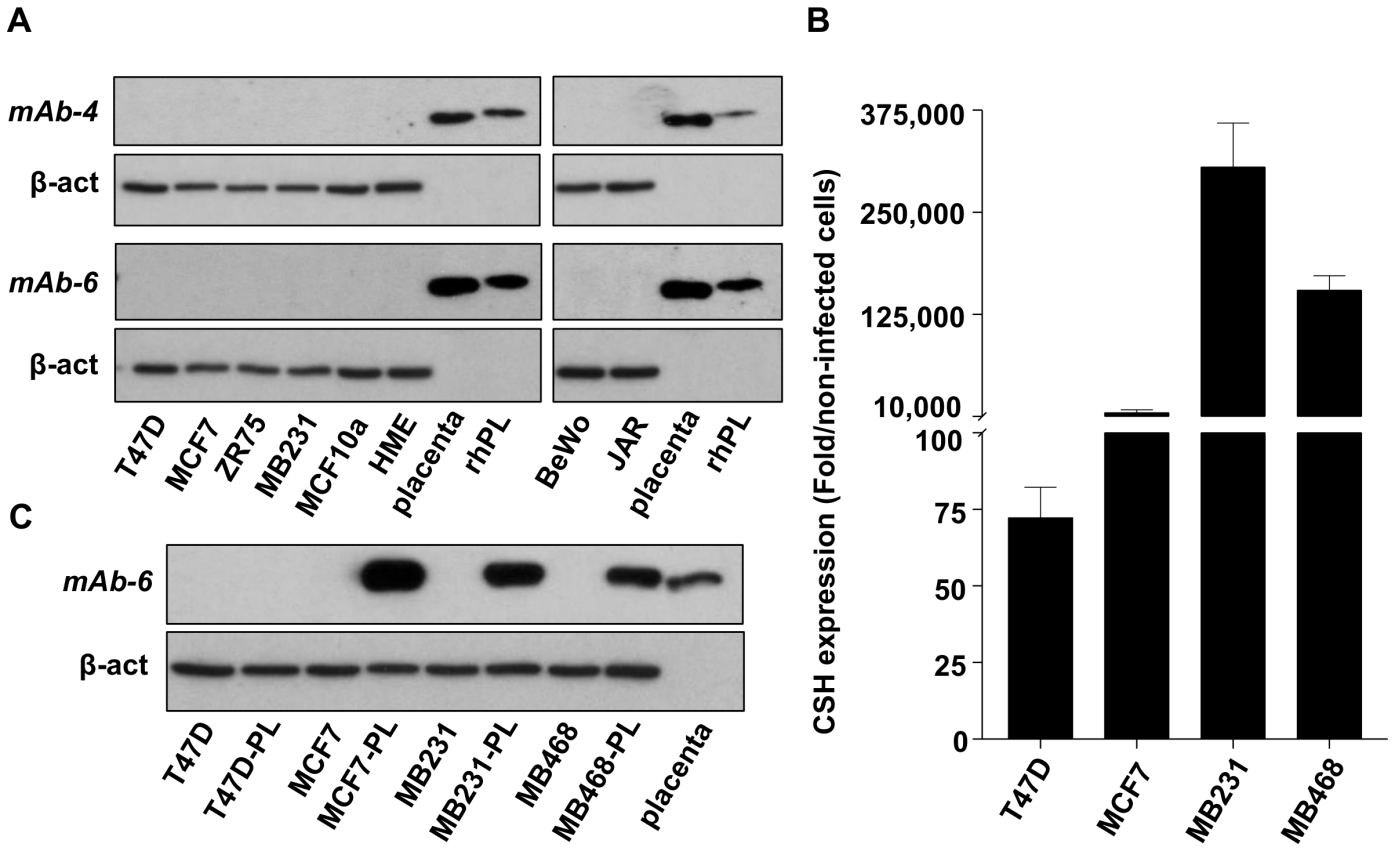
Detection of hPL protein in overexpressing, but not in control breast cancer cells by mAb-4 and mAb-6 monoclonal antibodies. (A) Whole cell lysates from breast cancer cells, non-tumorigenic breast epithelial cells and choriocarcinoma cells were subjected to western blotting; hPL was detected using mAb-4 and mAb-6; see Figure 2 for other details. (B) Real-time PCR was used to compare overexpression of CSH mRNA in lentiviral-infected breast cancer cell lines. Values (means±SEM; N = 3) represent fold increases over corresponding non-infected cells. (C) Whole cell lysates from control and PL-overexpressing cells were subjected to western blotting. hPL was detected using mAb-6 under the same conditions as in (A).

Still uncertain about the identity of ‘hPL’, we decided to use rhPL to generate two additional mAbs (mAb-4 and mAb-6). Unlike previous antibodies, mAB-4 and mAb-6 failed to detect hPL in either breast cancer or choriocarcinoma cell lines, although they clearly detected rhPL as well as hPL in placental extract (Figure 5A). Although disappointing, this finding still raised the possibility that breast cancer or choriocarcinoma cells produced a modified hPL which could not be detected by these mAbs. To pursue this possibility, we generated lentiviral constructs containing *CSH1* variant 1 (the same variant found in breast cancer cells), and used these to infect four different breast cancer cell lines, followed by harvesting of both RNA and protein.

As shown in Figure 5B, most infected cells expressed extremely high amounts of *CSH1* mRNA. Western blotting, using mAb-6, revealed detection of a protein of the same size as that found in the placenta (Figure 5C). Interestingly, T47D cells, which had the lowest expression levels among the infected cells, did not produce detectable hPL protein. These observations led us to conclude that when forced to overexpress *CSH1* at very high levels, BCC can produce hPL protein of the correct size. This suggested that the larger protein band recognized by all other antibodies (see Figure 2), is unlikely a post-translationally modified hPL, because the infected cells did not appear to modify an overexpressed protein. Because neither mAb-4 nor mAb-6 were suitable for IHC, (as determined with placental tissue), we could not examine for immunoreactive hPL in either the placenta or breast carcinomas with these particular antibodies.

### Many antibodies against hPL are non-specific

**Figure 6:**
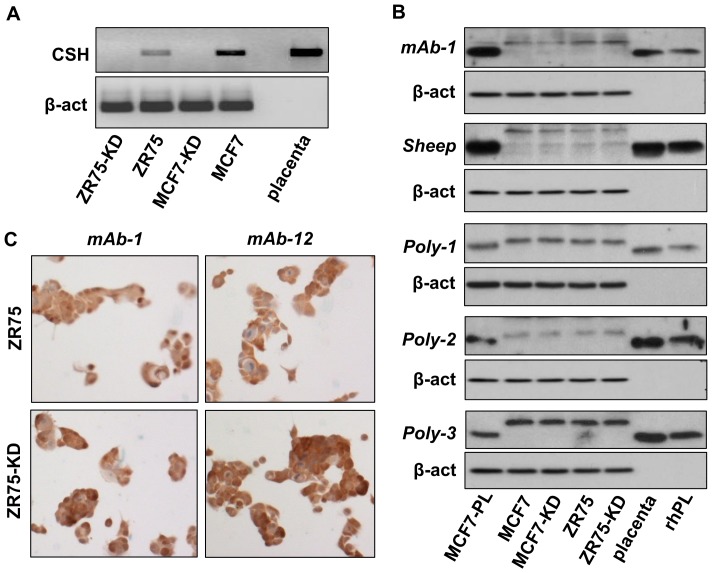
Multiple antibodies against hPL are non-specific. (A) Conventional PCR showing efficient knockdown (KD) of CSH in MCF7 and ZR75 following transfection with shRNA. The placenta was used as a positive control and size marker. (B) Western blots with five different antibodies showing an unchanged intensity of the bands previously believed to be ‘hPL’ in both control and knock-downed (KD) cells. (C) Similar intensity of immunocytostaining in control and knockdown ZR75 cells using mAb-1 and mAb-12.

Realizing that perhaps most hPL antibodies (except for mAb-4 and mAb-6) are non-specific, we sought further verification, and used shRNA to suppress CSH expression in MCF7 and ZR75 cells (MCF7-KD and ZR75-KD). As evident in Figure 6A, conventional PCR confirmed a very efficient suppression of CSH mRNA in these cells. Western blotting with multiple antibodies revealed that the ‘hPL’ band was, indeed, non-specific, as its levels were unchanged in spite of an effective suppression of the *CSH* gene (Figure 6B). In addition, both ZR75 control and ZR75-KD cells were plated in chamber slides, fixed and stained by immunocytochemistry to test the specificity of the antibodies used to detect hPL in breast carcinomas by IHC (see Figure 4). As seen in Figure 6C, the intensity of staining was not diminished when control and KD cells were compared, supporting the conclusion that neither mAb-1 nor mAb-12 were specific for hPL.

### hPL is not detected at the protein level in primary breast carcinomas using mAb-6

**Figure 7:**
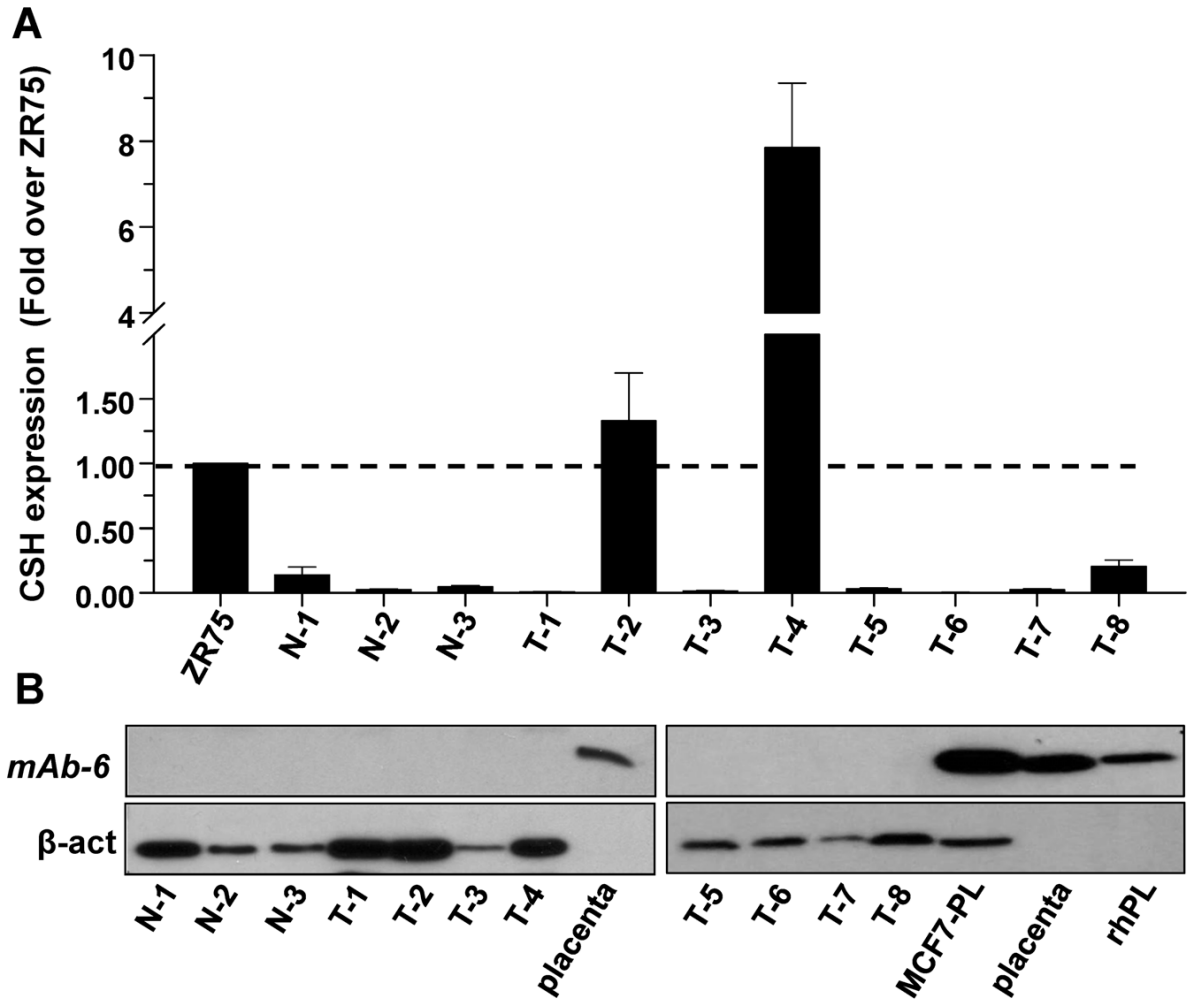
Expression of CSH mRNA in some primary breast carcinomas but undetectable hPL protein. (A) Comparison of CSH expression by real-time PCR in eight primary tumors (T1–T8) and three normal breast tissue samples (N1–N3). Data are presented as fold over ZR75 cells used for reference. (B) None of the above tissues show hPL protein, as determined in western blotting using mAb-6, which detected rhPL, placental hPL, and hPL in overexpressing MCF7 cells (MCF7-PL).

The inability of non-infected breast cancer cell lines to translate hPL mRNA into protein could be the consequence of a prolonged time in culture, whereas primary breast carcinomas may express this protein. Therefore, we used real-time PCR to compare *CSH* expression in ZR75 cells, three normal breast tissues (N-1 to N-3) and eight fresh frozen primary breast carcinomas (T-1 to T-8). Figure 7A shows that most samples had low to undetectable levels of CSH mRNA, with 2–3 tumors showing moderate levels. Upon using mAb-6 in western blotting, none of the tumors or normal breast tissues showed detectable levels of hPL protein (Figure 7B).

## Discussion

We are reporting that *CSH* is expressed at the mRNA level in breast cancer and choriocarcinoma cell lines, and in some primary breast carcinomas. The sequence of the cDNA product from BCC was identical to the placental *CSH1* variant. Several antibodies recognized a larger protein than hPL, leading us to postulate that it represents a post-translationally modified hPL. However, overexpression of hPL in breast cancer cell lines produced a product of identical size to that found in the placenta, indicating that breast cancer cells do not alter hPL post-translationally. We also ruled out the possibility that the hPL antibodies detected hPRL or hGH in BCC by probing the blots with antibodies specific to each lactogen. Two monoclonal antibodies which recognized rhPL, placental-derived hPL, and hPL produced by overexpressing cells, failed to detect the protein in unmodified breast cancer cells, in choriocarcinoma cells or in primary breast carcinomas. We conclude that most antibodies tested were, in fact, non-specific, and that *CSH* mRNA was not translated to protein in either breast cancer or choriocarcinoma cells.

We offer several explanations for the absence of hPL protein. One possibility is that *CSH* mRNA is indeed translated, but the protein is rapidly degraded. To this end, we treated BCC with increasing amounts of the proteasome inhibitor MG132 and examined for hPL protein by western blotting, using mAb-6. As a positive control, we used estrogen receptor alpha (ERα) protein. While ERα protein levels in BCC increased by MG132 treatment, hPL protein was still undetectable (data not shown). These data supported our conclusion that *CSH* mRNA is not translated into protein in breast cancer. In fact, several studies have shown discordance between mRNA and protein levels, particularly in human cancers [22]–[25]. Studies of global mRNA and protein levels in mammalian cells similarly find little correlation or predictive value [26]–[28].

Notably, overexpression of *CSH1* in BCC resulted in detectable hPL protein only when mRNA concentrations were extremely high. This suggests the existence of a post-transcriptional inhibitory mechanism(s) which is overwhelmed by large amounts of the message. Several mechanisms are known to decrease the efficiency of translation, including: a) short upstream open reading frames (uORFs), b) secondary structures in the 5′ untranslated region (UTR), and c) targeting by microRNAs (miRNAs). Many human and mouse transcripts contain at least one uORF, and its presence can decrease protein levels nearly 60% while only reducing mRNA levels 5% [29]. Complimentary base-pairing in the 5′ UTR can lead to formation of stable secondary structures that inhibit translation by interfering with ribosome scanning or by serving as recognition sites for inhibitory binding proteins. The *CSH* genes can utilize multiple origins of transcription in human placenta, producing mRNAs with variable 5′UTRs that have an identical coding sequence but may be translated at different rates [30].

MicroRNAs are non-coding RNAs which control translation of specific mRNAs by base pairing to sequences within target genes [31]. The miRNAs recruit the RNA-induced silencing complex (RISC), which can suppress translation of the message. Although miRNA are best known to target mRNA for degradation, they also mediate translational repression in the absence of mRNA decay. When conditions become favorable, these mRNAs may be efficiently translated [32]. If *CSH* mRNA is targeted by one or more miRNAs in breast cancer and choriocarcinoma cell lines, it does not appear to be degraded as a result, as the mRNA levels remain high despite lack of detectable protein.

All the polyclonal antibodies and two of the monoclonal antibodies examined in this study recognized the antigen they were made against, but also detected a 2–3 kDa larger protein which was confirmed not to be hPL since it was unchanged after suppression of *CSH* mRNA to undetectable levels. This protein must have considerable homology to hPL, and it would be interesting to find whether it also shares functional homology with hPL.

Pre-absorption of antibodies by the antigen is commonly used to verify antibody specificity. While this approach is widely practiced, it does not preclude cross-reactivity with other proteins. Indeed, a recent report which re-examined multiple antibodies used to measure the prolactin receptor in human breast cancer, concluded that most of the antibodies cross-reacted with other proteins such as cytokeratin 18 [33]. Our studies emphasize the need for a vigorous antibody validation, including gene suppression and/or overexpression whenever possible, when studying protein expression.

## Conclusions

We have provided evidence that the presence of *CSH* mRNA does not ensure detectable hPL protein in breast cancer and choriocarcinoma cell lines and in primary breast tumors. Previous investigators reporting detection of hPL protein in breast cancer may have been misled, as we were, by the presence of *CSH* mRNA in their samples, as well as the use of non-validated antibodies. Nonetheless, the mechanism by which cancer cells repress the translation of hPL protein is intriguing and deserves further investigation.
